# Correlation between Bone Mineral Density and Progression of Hip Osteoarthritis in Adult Men and Women in Bulgaria—Results from a 7-Year Study

**DOI:** 10.3390/life13020421

**Published:** 2023-02-02

**Authors:** Lyubomir Sapundzhiev, Tanya Sapundzhieva, Martin Mitev, Kiril Simitchiev, Anastas Batalov

**Affiliations:** 1 Department of Propedeutics of Internal Diseases, Medical Faculty, Medical University of Plovdiv, 4001 Plovdiv, Bulgaria; 2 Rheumatology Department, University Hospital ‘Pulmed’ Plovdiv, 4002 Plovdiv, Bulgaria; 3 Department of Analytical Chemistry and Computer Chemistry, Faculty of Chemistry, University of Plovdiv, 4001 Plovdiv, Bulgaria; 4 Rheumatology Clinic, University Hospital ‘Kaspela’, 4001 Plovdiv, Bulgaria

**Keywords:** bone mineral density, osteoarthritis progression, subchondral bone remodeling

## Abstract

Changes in clinical presentation, radiographic progression (RP), bone mineral density (BMD), bone turnover (BT), and cartilage turnover (CT) markers were compared in two groups of patients with hip osteoarthritis (HOA) over a period of 7 years. Each group consisted of 150 patients, including a control group on standard-of-care therapy (SC) with simple analgesics and physical exercises, and a study group (SG) on standard-of-care therapy supplemented by vitamin D3 and intravenous administration of zoledronic acid (5 mg) yearly for 3 consecutive years. Patient groups were homogenized regarding the following: (1) radiographic grade (RG), including 75 patients with hip OA RG II according to the Kellgren–Lawrence grading system (K/L), and 75 with RG III on K/L; (2) radiographic model (RM), as each of the K/L grades was subdivided into three subgroups consisting of 25 patients of different RMs: atrophic (‘A’), intermediate (‘I’), and hypertrophic (‘H’); (3) gender-equal ratio of men and women in each subgroup (Female/Male = 15/10). The following parameters were assessed: (1) clinical parameters (CP), pain at walking (WP-VAS 100 mm), functional ability (WOMAC-C), and time to total hip replacement (tTHR); (2) radiographic indicators(RI)—joint space width (JSW) and speed of joint space narrowing (JSN), changes in BMD (DXA), including proximal femur (PF-BMD), lumbar spine (LS-BMD), and total body (TB-BMD); (3) laboratory parameters (LP)—vitamin D3 levels and levels of BT/CT markers. RV were assessed every 12 months, whereas CV/LV were assessed every 6 months. Results: Cross-sectional analysis (CsA) at baseline showed statistically significant differences (SSD) at *p* < 0.05 in CP (WP, WOMAC-C); BMD of all sites and levels of CT/BT markers between the ‘A’ and ‘H’ RM groups in all patients. Longitudinal analysis (LtA) showed SSD (*p* < 0.05) between CG and SG in all CP (WP, WOMAC-C, tTHR) parameters of RP (mJSW, JSN), BMD of all sites, and levels of CT/BT markers for all ‘A’ models and in 30% of ‘I’-RMs (those with elevated markers for BT/CT at baseline and during the observation period). Conclusion: The presence of SSD at baseline (‘A’ vs. ‘H’) supported the thesis that at least two different subgroups of HOA exist: one associated with ‘A’ and the other with ‘H’ models. D3 supplementation and the intravenous administration of bisphosphonate were the treatment strategies that slowed down RP and postponed tTHR by over 12 months in the ‘A’ and ‘I’ RM with elevated BT/CT markers.

## 1. Introduction 

During the course of osteoarthritis (OA) of weight-bearing joints, articulate cartilage (AC) and subchondral bone (SB) act as one functional unit (osteochondral junction (OCJ)) against the altered biomechanical load [1,2,3,4]. 

SB is an composite term that includes the subchondral bone plate, the underlying trabecular bone, and the bone marrow space. The vertical portions of the arcades of collagen type II pass through it, anchoring the articular cartilage to the SB. In response to changed biomechanics, SB reacts with an increased turnover, leading to the accumulation of osteoid substance (sclerosis), in parallel with decreased mineralization due to the production of abnormal trimeric collagen, which has a low affinity for calcium. Thus, the process of thickening of the SB, known as eburnation, is the result of increased material density, together with decreased mineral density, increased porosity, and effacement of the cortical plate and the underlying trabecular bone. Besides eburnation, SB remodeling comprises the formation of subchondral bone cysts, new bone osteophytes, and changes in bone marrow (bone marrow lesions (BML)), detected with magnetic resonance imaging (MRI) and contrast-enhanced MRI (CE-MRI) [1,2,3,4]. The cytokines-mediated ‘cross-talk’ between chondrocytes and bone cells contributes to OA pathogenesis [2,3,4,5]. An alteration in the composition or structure of any of the individual components of the OCJ can initiate the pathogenetic processes leading to OA. In particular, in MRI studies, cartilage degeneration has been proven to be preceded by subchondral bone lesions (SBL), suggesting a key role for this mechanism in the pathogenesis and progression of OA, as well as in the formation of ectopic bone and osteophytes [3,4,5,6,7,8].

The changes in SB can be analyzed using parallel analyses of standard radiographic images (bone geometry, RG, and RM of HOA), MRI/CE-MRI (synovitis, BML, SBL), and BMD changes using DXA and/or quantitative computed tomography (QTC), with the latter assessing femoral strength using software for hip structural analysis. Histological sampling and analysis of BT/CT markers add further value to the imaging data. 

Over the past 40 years, researchers have tried to determine the relationship between BMD and the progression of clinical and morphological changes in HOA [9,10,11,12,13,14,15,16,17,18,19,20,21,22]. However, most studies have reported somewhat contradictory data, such as an association between HOA and increased or decreased BMD, both on a local level (PF-BMD) [10,11,15,18,22] and in the whole body (TB-BMD) [12,13,14,16,17,18], including patients with advanced HOA waiting for THR [19,20,21]. In the cited studies [9,11,12,13,14,15,16,17,18,19,20,21,22], the effect of several important factors was not taken into account. Despite the fact that CP, RP, and LP have been linked to the type of radiographic model (RM) (‘A’ vs. ‘I’ vs. ‘H’), this relationship was not factored into the research designs [10,23,24,25,26,27]. Furthermore, the inclusion of patients from a wide age range (35–85 years) does not allow control of secondary HOA. Patients aged 35 to 50 typically represent ‘H’ models of HOA, which are characterized by mild forms of hip dysplasia [28]. Gender-related differences (e.g., presence of SSD) in CP, mJSW, BMD, D3, and BT/CT markers between men and women of the same age, the same BMI, and the same RG/RM) have also been overlooked [29,30,31,32,33,34,35,36,37]. 

The current study aimed to investigate the relationship between BMD, microarchitecture, and SB remodeling, and the progression of HOA. For this purpose, a study group of HOA patients who received the standard-of-care treatment together with vitamin D3 supplementation [38] and intravenous administration of zolendronic acid (an anti-resorptive drug) [39] was compared to a control group of HOA patients receiving the standard-of-care therapy. To control for confounding factors, the RMs of HOA, gender differences, and the exclusion of all types of secondary HOA were taken into consideration when designing the study.

## 2. Methods and Materials 

This was a single-center, observational, randomized, double-blinded, and controlled study (DBRCT) that was conducted over a 7-year period (2014–2022) at the outpatient clinic of a rheumatology department. Each patient signed an informed consent form, approved by the Ethics Committee of the University Hospital “Pulmed”. The study was approved and registered at the Regional Health Inspectorate under the section “Radiation Control” with No. XI-214/06.07.2011 and by the Bulgarian Drug Agency with No. KИ-109-3-0009/12.01.2014.

### 2.1. Patients

The study included 300 participants in two groups, each consisting of 150 SG/CG patients, of whom 75 were RG-II K/L and 75 were RG-III K/L. Each RG was subdivided into three subgroups of 25 patients from different RMs (‘A’, ‘I’, ‘H’) with a fixed female to male ratio of 15/10 in each subgroup. In the SG group, in addition to the standard-of-care therapy, which included a simple analgesic (paracetamol—of up to 2.0 g/24 h) and physical exercises, the patients were also given the following: (1) vitamin D3 once daily, in the form of soft capsules ALPHA D3^®^ 1 μg, each containing 1 mcg. Alfacalcidol, Teva Pharmaceutical Industries Ltd., Tel Aviv, Israel (target level of 60 ng/mL, reference range 20–120 ng/mL); (2) ZA 5 mg/yearly (Aclasta^®^ 5 mg/100 mL Infusion, Novartis India Ltd, Mumbai, Maharastra, India.) for 3 consecutive years. 

#### Inclusion and Exclusion Criteria

The following inclusion criteria were applied: (1) a diagnosis of primary, one or double-sided HOA according to the ACR criteria [40]; (2) symptomatic HOA (WP by VAS ≥39/100 mm [41]), WOMAC-A ≥6/20, WOMAC-C ≥30/68 [42]); (3) radiographically confirmed HOA (RG II-III according to K/L classification [43]); (4) signed informed consent. 

Patients with the following characteristics were excluded from the study: (1) secondary HOA; (2) severe deviations in the weight-bearing axis; (3) presence of synovitis or effusion; (4) signs of rapidly progressing HOA in ‘A’-RM; (5) any intraarticular treatment or treatment with sulfate sugars, biocollagen, hyaluronic acid, diacerein, or avocado and soybean unsaponifiables within the 6 months prior to the baseline visit; (6) age above 60 and below 70; (7) body mass index (BMI) less than 21 kg/m^2^ or more than 28 kg/m^2^; (8) poorly controlled internal diseases, including hypertension, diabetes, cardiovascular, and cerebrovascular disease.

The age restrictions were introduced in order to exclude mild hip dysplasia (clinical presentation before 55 years of age) [28], the effect of hormonal changes in the early menopause (50–55 years) over SB [34], and the high rate of HOA in people over 60 years of age [18,22], as well as due to data regarding life duration (decreasing the chance of successfully finishing the 7-year follow-up period). The restriction in BMI and severe deviations in the weight-bearing axis were introduced in order to eliminate the effect of these variables on the RP of HOA [26,27]. The need for a fixed gender distribution in the patient groups was discussed previously [29,30,31,32,33,34,35,36,37].

### 2.2. Study Design 

The sample size of each patient group and subgroup was calculated using the methodology described by J. Wittes [44], considering a 2% possible loss of patients in the treatment groups, as compared to 1% in the control group. Restricted block randomization [45] was applied during the screening and selection process, resulting in the formation of the following six blocks: K/L-II’A’; K/L-II’I’; K/L-II’H’; K/L-III’A’; K/L-III’I’; and K/L-III’H’. From each block of 50 patients, subgroups with a fixed female to male ratio (15/10) were randomly generated using a computer program (Table 1). 

#### Blinding

The rheumatologists performing the clinical follow-up were blinded to the group types and numbers, as well as the laboratory and radiographic (DXA and X-ray) follow-up results. The medication was administered by the hospital pharmacist, who knew the randomization group and number but was blinded to the clinical and radiological data. The radiologists were blinded to the design, randomization group and number, and clinical data of the patients. The study coordinators (external, non-medical personnel provided by the Bulgarian Drug Agency) were responsible for contact with the patients (calling for the visits) and knew which randomization number corresponded to which patient, but were blinded to the clinical and radiological data of the patients. Only the principal monitor (external medical personnel provided by the Bulgarian Drug Agency) had access to the randomization group numbers and all data from CP and RI, but they did not know the patients. A summary of the research design, including the patient groups, methods, follow-up, and analyses, is presented in Table 1.

### 2.3. Physical Examination

Patient physical examinations and recording of the clinical data were performed by a board-certified rheumatologist. At each patient visit, the following factors were evaluated: vital signs, height, weight (BMI), walking pain (VAS-100 mm) [41], functional ability (WOMAC-C) [42], the presence of adverse events, and quality of life (SF-36 and PtGA) [46]. Treatment responses by OMERACT-OARSI set of responder criteria [47] and minimal clinically important improvement (MCII) [48] were also assessed.

### 2.4. Radiological Examinations

**Figure 1:**
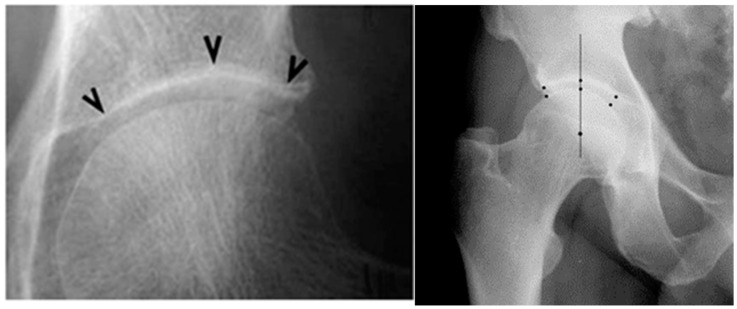
Measurement of JSW at three points according to the Barcelona consensus (**left image**) [29], and in a patient from our study (**right image**).

*The radiographic images* were taken in an upright weight-bearing position, anterior–posterior projection, with a slight (15–20 degrees) inner rotation of the feet, which was ensured by using a ‘V ‘-shaped pad placed 100 cm away from the source, with a perpendicular ray, focused on 4 cm above the symphysis. The following parameters were assessed: RG on K/L grade [37]; RM— ‘H’, ‘I’, ‘A’; JSW—the measurement of each joint was performed manually using software for measuring distances in digital radiographic images at three points: superolateral, apical, and superomedial (Figure 1). For the statistical analyses, the mean value of the three distances was used (mean joint space width—mJSW) and the annual speed of JSN-mm/year (JSNM12 = mJSW-M0 – mJSWM-12), according to the recommendations of the 2004 Barcelona Consensus Group [29].

**Figure 2:**
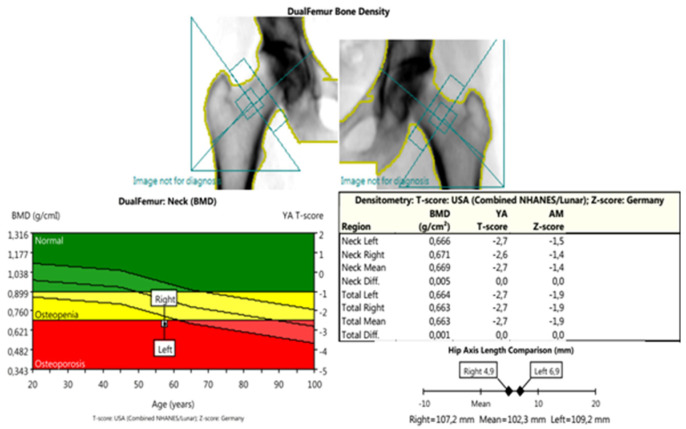
DXA measurements: dual femur—this scan was used to obtain the BMD parameters: femoral neck (FN-BMD); femoral head (FH-BMD); total hip BMD (TH-BMD); parameters of bone geometry (HAL—hip axis length; NSA—neck shaft angle; MNW—minimal neck width; CSA—cross-sectional area; CSMI—cross-sectional moment of inertia; FN-CT—cortical thickness of femoral neck; FS-CT—cortical thickness of femoral shaft; SM—section modules; BR—buckling ratio) and also for the comparisons between the mentioned parameters—left/right (target/non-target) joint.

**Figure 3:**
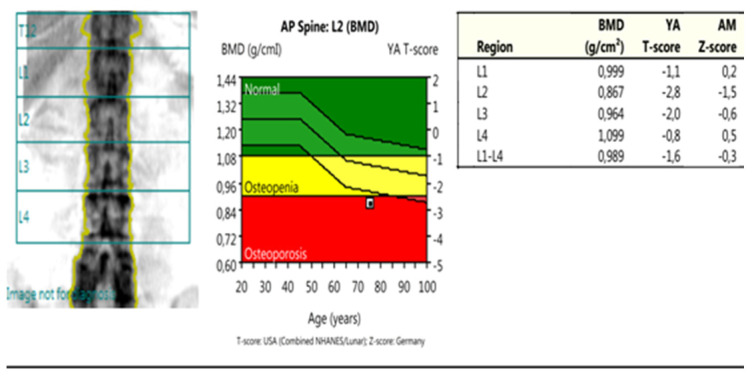
DXA measurements: anterior posterior spine—this scan was used to obtain the BMD parameters from the lumbar spine (LS-BMD), both from the individual vertebrae (L1; L2; L3; L4) and the total score (L1–L4).

**Figure 4:**
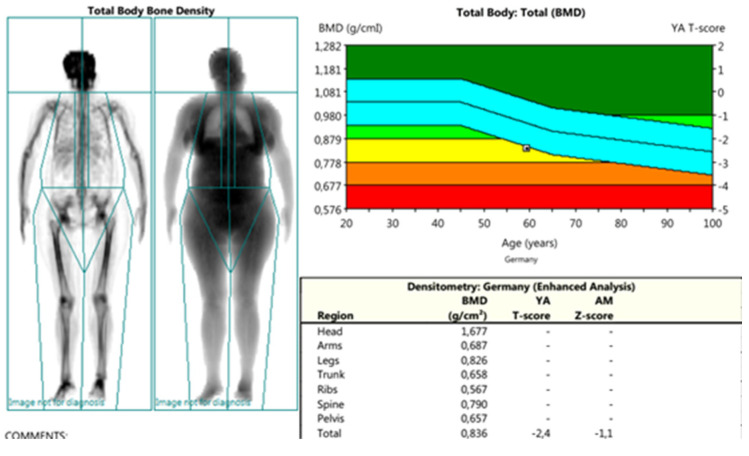
DXA measurements: total body—this scan was used to obtain the BMD parameters from the different regions (head-BMD; arms-BMD; legs-BMD; trunk-BMD; ribs-BMD; spine-BMD; pelvis-BMD) and a total body score (TB-BMD).

*DXA* measurements were conducted using *Lunar Prodigy Primo-en CORE, version 17,* according to the methodology of ISCD, including recommendations for calibration, measurement, and interpretation of results from 2015 [30]. The following parameters were assessed: PF-BMD; LS-BMD; TB-BMD (proximal femur, lumbar spine, total body); and parameters for bone geometry for HSA: HAL (hip axis length); NSA (neck shaft angle); CSA (cross-sectional area); CSMI (cross-sectional moment of inertia); MNW (minimal neck width); FN-CT (cortical thickness of the femoral neck); FS-CT (cortical thickness of femoral shaft); SM (section modulus); BR (buckling ratio) (Figure 2, Figure 3 and Figure 4). 

The measurements and interpretation of the results from the radiographic and DXA investigations were conducted by two separate certified by ISCD radiologists, who were blinded to the design and clinical and laboratory data, and with very good inter-reader reliability (intraclass correlation coefficient ICC of 0.918, 95% CI: 0.846–0.960) and PABAK (prevalence-adjusted and bias-adjusted kappa) values for X-ray/DXA reading of 0.860 and 0.880, respectively.

### 2.5. Biochemical Analyses

The biochemical analyses were performed and interpreted at the certified laboratory of the University Hospital ‘Pulmed’ in Plovdiv, Bulgaria. The serum levels of several markers were assessed, including

○ 25-hydroxy vitamin D (25-OH-D)—chemiluminescent immunoenzymatic assay (CLIA (reference range 20–120 ng/mL)) [31]
○ β-beta-isomerized carboxy-terminal cross-linking telopeptide of type I collagen (CTX-I, in our country - β-Cross Laps), a product of the break-down of collagen type I by the osteoclasts, with the former comprising 90% of the organic bone matrix, a marker of bone degradation [31], CLIA methodology (reference range: men (>60 years old) <0.7 ng/mL; women >60 years old (postmenopausal) <0.9 ng/mL).
○ Urine C-terminal crosslinking telopeptides of collagen type II (CTX-II)—a marker of CT [32,33,34,35,36,37] (competitive ELISA, Cartilaps, IDS, Boldon, UK, (reference range 129 and 345 ng/mmol Cr), with intra- and inter- assay CVs below 8% and 10%, respectively).

The concentration of CTX-II (ng/L) was standardized to the total urine creatinine (mmol/L), and the units for the corrected CTX-II concentration were ng/mmol [33]. Quantitative detection of creatinine in urine was performed using a Human Creatinine ELISA Kit, Chongqing Biospes Co., Ltd. (Catalog No: BYEK2883). The corrected concentration of uCTX-II for urinary creatinine was calculated using the formula: corrected uCTX-II (ng/mmol) = 1000 × uCTX-II (ug/L)/urinary creatinine (mmol/L). 

### 2.6. Follow-Up

The following parameters were assessed for the patients in both groups (CG and SG): RV (JSW/JSN; BMD-DXA); CV—(WP; F - WOMAC-C; PtGA; tTHR); LV—levels of vitamin D3 and levels of BT/CT markers. RV were assessed every 12 months, whereas CV and LV were assessed every 6 months. 

### 2.7. Statistical Analyses

The data were analyzed using Statistical Package for the Social Sciences (SPSS) version 21. The results were presented as numbers and percentages (%) for the qualitative variables and as medians with interquartile ranges (IQRs) for the continuous variables. For the detection of statistically significant differences (SSDs), Mann–Whitney and Kruskal–Wallis non-parametric tests were used. If SSD was present, post hoc multiple comparisons with Dunn–Bonferroni were performed. Pearson correlation analysis and multiple logistic regression were used to analyze the effect of BMD and bone geometry markers on radiographic progression. All statistical tests were two-tailed and performed at a level of significance (α) of 0.05 and a power of 80%. Exact *p*-values were used to interpret the results, with a *p* < 0.05 meaning a statistically significant change.

## 3. Results

### 3.1. Results from the Cross-Sectional Study

There were no significant differences in the values of the clinical, laboratory, and radiographic parameters at baseline between the SG and CG groups in all three models (Table 2).

**Figure 5:**
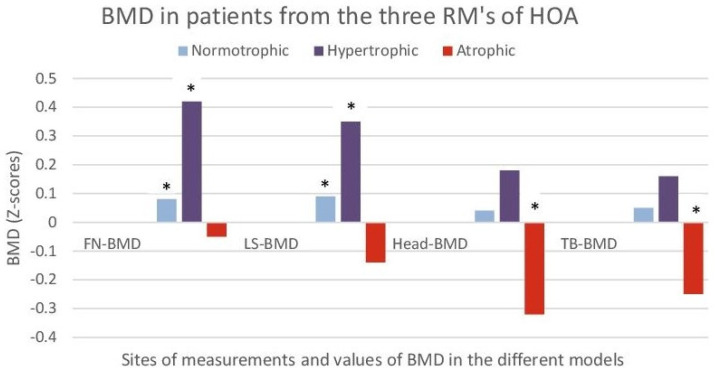
Bone mineral density (BMD) in patients from the three RMs of HOA (hypertrophic; normotrophic and atrophic). The results are shown as Z scores of femoral neck (FN), lumbar spine (LS), scull (Head), and total body (Total) for each RM. Z scores were used to compare to controls without HOA after standardization for gender, age, and BMI. *- a statistically significant difference (*p* < 0.05) when comparing to controls without HOA after standardization for gender, age, and BMI.

All ‘A’ models from both RGs of the two groups had decreased BMD at all measurement sites (PF-BMD; LS-BMD; TB-BMD) and 70% of them met the ISCD criteria [30] for osteoporosis. On the other hand, all ‘I’ and ‘H’ models were with normal (‘I’) or slightly increased (‘H’) local BMD (*p* < 0.05), as compared to the controls without HOA (Figure 5).

**Figure 6:**
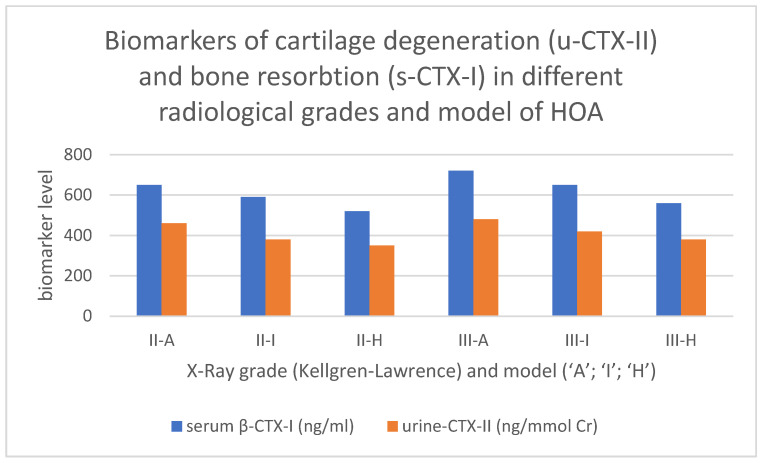
Values of markers for bone (serum β-CTX-I) and cartilage (urine CTX-II) turnover in the different RG/RM of HOA. Serum β-CTX-I—beta-isomerized carboxy-terminal cross-linking telopeptide of type I collagen in nanogram per milliliter; urine CTX-II—C-terminal crosslinking telopeptides of collagen type II, presented as corrected concentrations of uCTX-II for urinary creatinine concentration, as ng/mmol Cr.

All ‘A’ models had increased levels of CT or BT markers as compared to the ‘I’ model. The latter showed increased CT/BT markers as compared to the ‘H’ model, *p* < 0.05 (Table 2 and Figure 6).

The comparison between ‘A’ and ‘H’ models showed statistically significant differences in CP (*p* < 0.01), the levels of BT/CT markers, and BMD and bone geometry markers (a wider femoral neck and increased FSI, *p* < 0.05), and no significant differences in mJSW. The comparisons of ‘H’ vs. ‘I’ and of ‘I’ vs. ‘A’ showed some differences, but they were not statistically significant (*p* > 0.05), even at the interim analyses (Table 3).

### 3.2. Results from the Longitudinal Study

Serious adverse effects were not observed in either group, and there was no loss of patients during the 7-year follow-up period. Transient myalgia (2–6 h with spontaneous resolution) after the intravenous administration of ZA was observed in 46 patients (31%) from the SG group.

**Figure 7:**
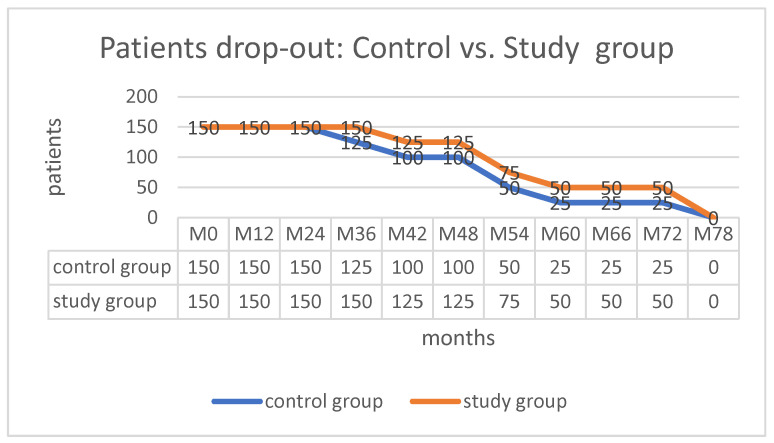
Follow-up data.

The loss of whole subgroups was observed at the following times: M36—CG-III’A’; M42—CG-III’I’/SG-III’A’; M54—CG-III’H’ + II’A’ /SG-II’H’ + III’I’; M66—CG-II’I’/SG-II’A’; and M78—CG-II’H’/SG-II’H’+II’I’ (Figure 7). This was an expected result of HOA’s natural and therapy-modified evolution, rather than the loss of subjects. The final clinical and radiographic investigations were completed, with a clear path toward conversion to total hip replacement (THR) and the possibility of follow-up.

**Figure 8:**
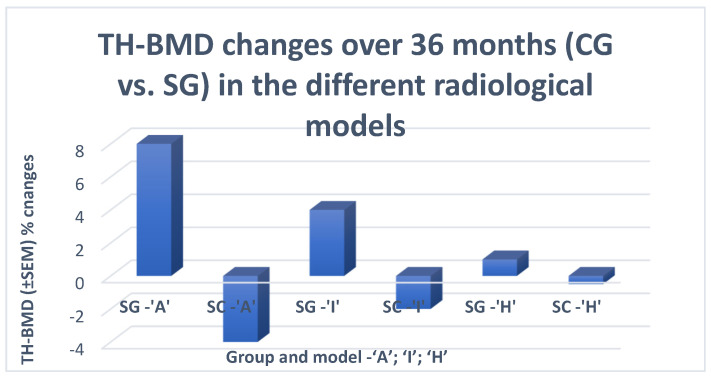
Change in BMD for a period of 36 months in the control (SC) and treatment (SG) groups. TH-BMD—total hip bone mineral density. The levels are presented as standard error of the mean (SEM) percentage changes, in order to present the characteristics (changes in BMD) of the sample data and allow comparisons of how much discrepancy was likely to appear in the sample mean as compared to the population mean.

In the CG group, the natural evolution of HOA did not demonstrate unexpected results. While the CT and BT markers were constantly increasing, CP (WP and F) worsened over time, along with advances in morphological changes (mJSW) and decreases in BMD at all measurement visits (Figure 8 and Table 4).

In the SG group, a delay in the decrease in BMD followed by a transient increase in BMD was observed in all RMs from both RGs, albeit at different rates: at month 12 (M12) in the ‘A’ and ‘I’ models and at M36 in the ‘H’ model. This increase was followed by a rapid decrease in BMD, starting from M48 (12 months after the last administration of ZA), at the same rate of change as in the SC group (Table 4). BMD changes were accompanied by similar changes in the levels of BT/CT markers, namely a decrease followed by an increase after M48 at the same rate as in the CG group. The described changes in CT/BT and BMD were accompanied by transient but statistically significant differences in the values of CP and RI (SG vs. CG). 

### 3.3. Within Group Comparisons

At baseline (M0) in both patient groups (SC and SG), a significant difference existed between the endmost RMs (‘H’ vs. ‘A’) in CP (WP/F). No significant differences were found in mJSW, including the end RMs. During the follow-up, at points M12 (CP’s/ JSN) and M36 (mJSW), significant differences were observed between all RMs of the SC group (Table 5 and Table 6).

The within-group comparisons in CP in the SG sample (‘H’ vs. ‘I’) did not show significant differences throughout the study period (K/L-II M78; K/L-III –M54). On the other hand, the comparisons of ‘I’ vs. ‘A’ revealed significant differences at M60 (K/L-II) and at M36 (K/L-III) (Table 5). Regarding the radiological indicators, the comparisons in the SG showed no significant differences (‘H’ vs. ‘I’ vs. ‘A’) in the slow progression stage (K/L-II) until the end of the follow-up. In the rapidly progressing stage (K/L-III), the intragroup comparisons of ‘H’ vs. ‘I’ vs. ‘A’ showed no significant differences in JSN, despite the presence of differences in mJSW (M12/M36), suggesting a slowing down of the speed of JSN (Table 6). 

Therapeutic responses, according to the OMERACT-OARSI set of responder criteria [49] and Tubach F. (minimal clinically important improvement –MCII) [50] were registered in ‘A’ and 30% of ‘I’ RMs from both radiological grades, K/L-II and III (CG vs. SG) in CP (WP; WOMAC-C; PtGA). These responses were first detected at month six after the first application of ZA and persisted until the sixth (OMERACT-OARSI set) or twelfth month (MCII) after the last ZA administration (Table 4). 

### 3.4. Between Group (SC vs. SG) Comparisons

The first occurrence of significant differences in JSN between the two groups (SG and CG) was detected at month 12 after the first application of ZA in the ‘A’ models of both RGs and in 30% of ‘I’ models, namely those with elevated markers for BT and CT at baseline and during the observation period. These differences were accompanied by analogous ones in mJSW with a later onset at M36 and persisted during all follow-up visits, until the end of the study (Table 7). 

Statistically significant differences between the study and control groups were observed in the ‘A’ and ‘I’ models from both RGs regarding the length of time before THR had to be performed. Due to the longer natural evolution of HOA in the K/L-II grade (K/L-II’A’ vs. K/L-III’A’) these differences were highly significant (*p* < 0.001) (Table 8). 

By using multiple logistic regression, the observed changes in BMD (CG vs. SG) were identified as the main reason (Table 9) for the observed changes in CP and RP. The decrease in their values by one standard deviation (SD) was associated with an accelerated progression (OR = 6.561, *p* < 0.001; OR = 6.495, *p* < 0.001, respectively).

## 4. Discussion 

The methodology of computer-simulated models, which is widely used today [49,50], had been not developed in detail at the time of the current study’s planning (2011–2013), resulting in the planning and execution of an experimental study instead of a simulation study.

The gender-related differences in the levels of CB and CT markers and radiological indicators (mJSW/BMD) in men and women with the same values of CP (WP/F), radiological grade, and model, are well-known and have been discussed in the literature [29,30,31,32,33,34]. However, these gender differences, as well as the differences in CP, levels of the CT-BT markers, and BMD between ‘A’ and ‘H’ models of the same RG, suggest that patient groups should be homogenized not only by RG, but also by RM, with a fixed ratio in the number of men and women in each group. Furthermore, the significant differences in CP, CT/BT markers, and BMD in the absence of corresponding changes in mJSW between ‘A’ and ‘H’ RMs of the same RG suggest that the value of the CP (WP/F) is dependent not only on mJSW (loss of AC), but also on the quality (changes) in the SB.

Differences in bone geometry, BMD, levels of BT/CT markers, and bone histomorphometry between the different RMs of HOA were reported in a number of studies [10,24,25,26]. ‘H’ models had a wider femoral neck, increased BMD at all measurement points, and increased FSI compared to ‘A’ models, which had a narrower femoral neck, decreased BMD at all measurement points, and decreased FSI. The levels of BT/CT markers showed delayed or accelerated bone resorption in the ‘H’ as compared to ‘A’ models [24,36], whereas histomorphometric studies of specimens from THR surgeries showed smaller bone volumes and thinner trabeculae in ‘A’ models [25]. 

One of the objectives of our study was to examine the relation between RM and BMD in the Bulgarian population and the treatment options available to delay RP in patients with different types of RM of HOA through vitamin D3 supplementation and intravenous administration of ZA (which causes a change in BMD).

The results from the comparison between bone geometry parameters, BMD, and the levels of serum CTX-I and urine CTH-II at baseline in our study were similar to those reported in other similar studies [10,24]. The abovementioned results (ours and from other studies) suggest the presence of at least two different types of HOA, associated with ‘A’ and ‘H’ models.

At baseline, within-group comparisons in both groups (SC and SG) showed significant differences between the endmost RMs (‘H’ vs. ‘A’) in CP (WP/F) and an absence of significant differences in mJSW, even between the endmost models.

During the follow-up, the natural evolution of the SC group led to the appearance of significant differences at M12 in the clinical parameters and JSN and at M36 in mJSW among all RMs. The absence of such differences (‘H’ vs. ‘I’) and their later onset (‘I’ vs. ‘A’ at M60 for K/L-II and at M36 for K/L-III) in the SG group could be attributed to the therapy-modified (D3 + ZA) evolution of the HOA. These factors suggest that the changes in BMD due to the anti-resorptive therapy equalized the rate of the radiographic progression of the models with reduced BMD (‘A’) to those with a normal (‘I’) or slightly increased (‘H’) BMD. 

At baseline, all ‘A’ models in both RGs in the two groups (SC and SG) had decreased BMD at all measurement sites (PF-BMD; LS-BMD; TB-BMD), all ‘I’ models had normal BMD, and all ‘H’ models had normal or slightly increased local BMD. 

The analysis of the BMD change over the follow-up period demonstrated that the BMD of the control group decreased, reaching the normal, or lower than the normal, BMD of patients without HOA (at all measurement sites) as early as the second (‘I’) or the third year (‘H’), which was accompanied by a decrease in vitamin D3 levels and an increase in serum CTX-I and urinary CTX-II. A possible explanation for the changes in vitamin D3, s-CTX-I, and u-CTX-II levels could be the decreased physical activity and lifestyle changes related to HOA progression. Nevertheless, the observed significant differences at baseline (CsA) in TB-BMD and head BMD (which are not related to the biomechanics in HOA), cannot be explained in this way, but rather by the presence of different types of HOA, as was discussed earlier. 

A possible explanation for the changes in vitamin D3, s-CTX-I, and u-CTX-II levels is the decreased physical activity and lifestyle changes related to HOA progression. Nevertheless, the observed significant differences at baseline (CsA) in TB-BMD and head BMD, which are not related to the biomechanics in HOA, cannot be explained in this way, but rather by the presence of different types of HOA, as was discussed earlier. 

The longitudinal significant differences in RP (JSN-M12/mJSW-M36) between the two groups (SC vs. SG), as well as in the tTHR between the ‘A’ and ‘I’ models of both groups, showed a clear relationship with the detected changes in BMD (FN-BMD; TH-BMD. This relation correlates by grade with the well-known impact of these parameters on the fracture risk. In our study, a decrease in FN-BMD and TH-BMD by 1 SD was associated with accelerated radiographic progression and increased fracture risk. 

To our knowledge, no other studies have examined the effect of intravenous ZA on the progression of HOA [51]. The only study evaluating the effect of a bisphosphonate administration on the progression of HOA used oral alendronate [52]. In the present study, we observed a significant improvement in VAS and WOMAC scores and BMD, with a parallel decrease in the values of sCTX-I and uCTX-II, but no significant delay in the progression of HOA, defined as a decrease in JSW >0.30 mm or conversion to total hip arthroplasty [52]. 

At the same time, a number of studies have analyzed the efficacy of ZA administration in reducing postoperative complications after THR [53]. There have also been a large number of studies similar to ours but on the efficacy of ZA administration in knee OA (KOA) [54]. Those KOA studies showed contradictory results, which, in addition to the failure of alendronate to slow the radiographic progression of HOA [52], are most likely due to the inconsistency of the study designs used; the discussed influence of the radiographic model and gender on the clinical and radiographic variables.

Considering the rates of the different RMs of HOA in the general population (60% ‘I’-RM; 30% ‘H’-RM; 10% ‘A’-RM) [23,24], and this study’s results, which showed that the treatment was effective in all ‘A’ models and in 30% of ‘I’ models (those with persistently elevated BP/CT), it can be extrapolated that 30% of patients with HOA would benefit from a combined treatment of D3 with ZA. At first glance, this percentage does not seem so high, but considering the prevalence of HOA among the population over 60 years of age, 0.1–0.2% of people in the general population would benefit from the treatment strategy used in our study.

With regard to when an anti-resorptive therapy in patients with ‘A’ models of HOA should be started, our recommendation is as early as possible. At baseline, all ‘A’ models of HOA had DXA scores of osteopenia or osteoporosis (70%) at all measurement sites and increased levels of BT and CT markers. 

All ‘I’ models with increased BT and CT markers had slightly reduced BMD at all measurement sites, as compared to the controls without HOA, without fulfilling the ISCD criteria for osteopenia [30]. However, within the two-year follow-up, these values changed and fulfilled the criteria for osteopenia in 100% of patients and for osteoporosis in 56% of them.

Our findings suggest that patients with ‘I’ models of HOA who have elevated BP and CT markers should be followed up on an annual basis. If a significant decrease in BMD is detected on two consecutive measurements, antiresorptive therapy should be started, even if the DXA scores do not meet the ISCD criteria for osteoporosis.

However, it is important to remember that all ‘H’ and ‘I’ models of HOA with normal BT and CT markers, which accounts for 70% of HOA patients, do not benefit significantly from anti-resorptive therapy.

Our study has several limitations that need to be addressed. One of them is the small number of bone (serum CTX-I) and cartilage (urinary -II) turnover markers, which could not adequately demonstrate the composite mechanism of the processes occurring at the osteochondral junction. Another limitation stems from the dual nature of the DXA investigation used for HSA, in the absence of parallel data from a three-dimensional imaging modality (QTC). Another area for improvement is the calculation of mJSW and JSN based on manual measurements of JSW at three points rather than MRI-based assessments. 

In conclusion, the significant differences in CP, BMD, bone geometry parameters, and levels of BT and CT markers observed at baseline between the ‘A’ and ‘H’ models support the hypothesis of the existence of two distinct subgroups of HOA. The one associated with ‘A’ models and the other with ‘H’ models exhibit specific characteristics of AC-breakdown, SB-remodeling, and clinical and radiological progression, and a different response to anti-resorptive drugs. In ‘A’ and ‘I’ models with increased BT and CT markers, D3 supplementation and intravenous administration of ZA constitute a treatment strategy that slows RP and delays tTHR for more than a year.

Given the limitations of our study and the significant socioeconomic impact of hip osteoarthritis, larger randomized controlled trials with an appropriate design, sample size, and duration should be conducted.

## Figures and Tables

**Table 1:** Research design: patient groups, methods, follow-up, indicators, and analyses.

Participants:	Methodology:	Analyses:
300 patients: 150 grade II K/L 150 grade III K/L Two groups: control (150 patients)/study (150 patients) Control group: *75 patients K/L-II* 25—‘A’-models (♀/♂ = 15/10) 25—‘I’-models (♀/♂ = 15/10) 25—‘H’-models (♀/♂ = 15/10) *75 patients K/L-III* 25—‘A’-models (♀/♂ = 15/10) 25—‘I’-models (♀/♂ = 15/10) 25—‘H’-models (♀/♂ = 15/10) Study group: *75 patients K/L-II* 25—‘A’-models (♀/♂ = 15/10) 25—‘I’-models (♀/♂ = 15/10) 25—‘H’-models (♀/♂ = 15/10) *75 patients K/L-III* 25—‘A’-models (♀/♂ = 15/10) 25—‘I’-models (♀/♂ = 15/10) 25—‘H’-models (♀/♂ = 15/10)	***Clinical assessment*** (every 6 months): ✓ Pain at walking (VAS 100 mm) ✓ WOMAC-C ✓ tTHR ***Laboratory assessment*** (every 6 months): ✓ D3 -levels ✓ s-CTX-I –levels ✓ u-CTX-II –levels ***X-Ray examination*** (every 12 months): ✓ radiographic grade (K/L) ✓ radiographic model (‘A’; ‘I’; ‘H’) ✓ mJSW ✓ JSN ***DXA-examinations*** (every 12 months): ✓ PF-BMD ✓ LS-BMD ✓ TB-BMD	(1) ***Cross-sectional analyses at baseline***: ✓ Intragroup comparisons between RM—‘H’ vs. ‘I’ vs. ‘A’ from the same RG, on the same treatment ✓ Intergroup comparisons—between relevant subgroups of SC vs. SG (2) ***Longitudinal analyses over the time of follow up***: ✓ Intragroup comparisons between RM—‘H’ vs. ‘I’ vs. ‘A’ from the same RG, on the same treatment ✓ Intergroup comparisons—between relevant subgroups of SC vs. SG (3) ***Multiple logistic regression***, to assess the effect of changes in BMD on radiographic progression.

K/L—according to Kellgren–Lawrence grading scale; ‘H’/’I’/‘A’—hypertrophic/intermediate/atrophic-radiographic patterns (models) of hip osteoarthritis according to the balance between the narrowing of the joint space and the osteophyte growth; VAS—visual analogue scale; WOMAC-C—WOMAC-function scale; tTHR—time to conversion to total hip replacement; D3—25-hydroxy vitamin D; s-CTX-I—serum-beta-isomerized carboxy-terminal cross-linking telopeptide of type I collagen; u-CTX-II—urine -C-terminal crosslinking telopeptides of collagen type II; mJSW—mean joint space width; JSN—joint space narrowing; BMD—bone mineral density; PF/LS/TB—proximal femur, lumbar spine, total body.

**Table 2:** Baseline characteristics of the control and study group.

Parameter ^#^	‘H’ Models	‘I’ Models	‘A’ Models	*p* *
Median ± IQR	Median (IQR)	Median (IQR)	Median (IQR)	CG vs. SG
Age K/L-II				0.997
Control group (SC)	65 (64–67)	64 (63–66)	62 (61–63)	
Treatment group (SG)	65 (64–67)	64 (63–66)	62 (61–63)	
Age K/L-III				
Control group	66 (64–68)	65 (63–68)	63 (62–64)	
Treatment group	66 (63–69)	65 (64–68)	63 (61–65)	
BMI K/L-II				0.995
Control group	25.5 (24–27)	24.5 (23.5–26.5)	24.0 (23.0–25.0)	
Treatment group	25.7 (23.7–27.7)	25.0 (23.0–27.0)	23.5 (22.5–25.0)	
BMI K/L-III				
Control group	26.5 (25.0–27.0)	26.0 (25.0–27.0)	24.5 (23.5–25.5)	
Treatment group	26.8 (25.0–27.8)	25.5 (24.5–26.5)	25.0 (24.0–26.0)	
Gender K/L-II				1.000
Control group	♀-15/♂-10	♀-15/♂-10	♀-15/♂-10	
Treatment group	♀-15/♂-10	♀-15/♂-10	♀-15/♂-10	
Gender K/L-III				
Control group	♀-15/♂-10	♀-15/♂-10	♀-15/♂-10	
Treatment group	♀-15/♂-10	♀-15/♂-10	♀-15/♂-10	
WP (VAS mm.) K/L-II				0.993
Control group	41 (40–42)	43 (42–44)	45 (42–48)	
Treatment group	41 (39–42)	43 (41–45)	45 (43–47)	
WP (VAS mm.) K/L-III				
Control group	48 (46–50)	50.5 (48.5–52.5)	53 (51–55)	
Treatment group	48 (45–51)	51 (49–53)	54(53–55)	
F (WOMAC-C) K/L-II				0.951
Control group	31.5 (30–33)	34.5 (33–36)	36.5 (35–38)	
Treatment group	32 (30–34)	35 (34–36)	37 (36–38)	
F (WOMAC-C) K/L-III				
Control group	37.5 (36–39)	40.5 (39–42)	42.5 (41–44)	
Treatment group	38 (36–40)	41 (39–43)	43 (41–45)	
25-OH-D (ng/mL) K/L-II				0.921
Control group	39.5 (33.5–45.5)	36.0 (30.5–41.5)	31.5 (27.5–33.5)	
Treatment group	39.0 (33.0–45.0)	35.6 (30.6–40.6)	31.0 (27.5–33.0)	
25-OH-D (ng/mL) K/L-III				
Control group	35.8 (31.8–39.8)	32.4 (29.4–35.4)	27.5 (25.5–29.5)	
Treatment group	35.5 (31.5–39.5)	32.0 (29.0–35.0)	27.0 (25.5–29.5)	
s-CTX-I (ng/mL) K/L-II				0.913
Control group	520 (460–600)	590 (510–670)	650 (600–700)	
Treatment group	525 (465–600)	594 (514–680)	655 (610–700)	
s-CTX-I (ng/mL) K/L-III				
Control group	560 (500–620)	645 (600–700)	715 (650–780)	
Treatment group	564 (510–620)	654 (604–700)	720 (660–790)	
u-CTX-II (ng/mmol Cr) K/L-II				0.912
Control group	346 (300–390)	378 (330–415)	456 (416–496)	
Treatment group	354 (304–394)	382 (334–420)	464 (420–500)	
u-CTX-II (ng/mmol Cr) K/L-III				
Control group	378 (318–438)	418 (350–478)	476 (426–526)	
Treatment group	384 (324–442)	423 (353–483)	484 (430–530)	
mJSW (mm.) K/L-II				0.956
Control group	4.5 (4.4–4.6)	4.4 (4.0–4.8)	4.3 (4.0–4.6)	
Treatment group	4.5 (4.3–4.7)	4.4 (4.1–4.7)	4.3 (4.1–4.5)	
mJSW (mm.) K/L-III				
Control group	3.7 (3.6–3.8)	3.5 (3.4–3.6)	3.3 (3.1–3.5)	
Treatment group	3.7 (3.5–3.9)	3.5 (3.3–3.7)	3.3 (3.0–3.6)	
AP spine L1–L4 BMD (g/cm^2^) K/L-II				0.948
Control group	1.3 (1.0–1.5)	1.1 (0.9–1.3)	0.8 (0.7–0.9)	
Treatment group	1.3 (1.1–1.5)	1.1 (0.8–1.3)	0.8 (0.6–0.9)	
AP spine L1–L4 BMD (g/cm^2^) K/L-III				
Control group	1.0 (0.8–1.2)	0.8 (0.5–1.1)	0.6 (0.5–0.7)	
Treatment group	1.0 (0.9–1.1)	0.8 (0.6–1.0)	0.6 (0.53–0.67)	
Total hip BMD (g/cm^2^) K/L-II				0.947
Control group	0.8 (0.6–1.0)	0.6 (0.5–0.7)	0.45 (0.39–0.51)	
Treatment group	0.8 (0.7–0.9)	0.6 (0.5–0.65)	0.45 (0.38–0.51)	
Total hip BMD (g/cm^2^) K/L-III				
Control group	0.7 (0.63–0.8)	0.5 (0.43–0.60)	0.35 (0.32–0.38)	
Treatment group	0.7 (0.61–0.8)	0.5 (0.41–0.61)	0.35 (0.33–0.37)	
Femoral neck BMD (g/cm^2^) K/L-II				0.946
Control group	0.9 (0.8–1.0)	0.7 (0.6–0.8)	0.5 (0.4–0.6)	
Treatment group	0.9 (0.7–1.1)	0.7 (0.62–0.78)	0.5 (0.43–0.57)	
Femoral neck BMD (g/cm^2^) K/L-III				
Control group	0.8 (0.67–0.9)	0.64 (0.6–0.68)	0.4 (0.36–0.44)	
Treatment group	0.8 (0.7–0.91)	0.63 (0.6–0.67)	0.42 (0.38–0.44)	
Total body BMD (g/cm^2^) K/L-II				0.945
Control group	1.5 (1.3–1.7)	1.4 (1.3–1.5)	1.3 (1.2–1.4)	
Treatment group	1.5 (1.4–1.6)	1.4 (1.2–1.6)	1.3 (1.1–1.5)	
Total body BMD (g/cm^2^) K/L-III				
Control group	1.4 (1.3–1.5)	1.3 (1.2–1.4)	1.2 (1.1–1.3)	
Treatment group	1.4 (1.2–1.6)	1.3 (1.1–1.5)	1.2 (1.0–1.4)	

^#^—Each study subgroup consisted of 25 subjects; *—The Kruskal–Wallis test was applied; WP—pain at walking; F (WOMAC-C)—WOMAC function scale; mJSW—mean joint space width; 25-OH-D—25-Hydroxy vitamin D in nanogram per milliliter; s-CTX-I—serum-beta-isomerized carboxy-terminal cross-linking telopeptide of type I collagen in nanogram per milliliter; u-CTX-II—urine-C-terminal crosslinking telopeptides of collagen type II, given as corrected concentration of uCTX-II for urinary creatinine concentration in ng/mmol Cr.

**Table 3:** Within-group comparisons (‘H’ vs. ‘I’ vs. ‘A’) of the values of CP, RI, and CT/BT markers in both groups.

X-Ray Grade												
K/L-II							K/L-III					
X-Ray Model	‘H’		‘I’		‘A’		‘H’		‘I’		‘A’	
Groups ^#^	SC	SG	SC	SG	SC	SG	SC	SG	SC	SG	SC	SG
WOMAC-C M0-Median (IQR)	31 (30–32)	32 (31–33)	33 (33–35)	34 (32–36)	36 (35–37)	36 (35–37)	38 (37–39)	38 (37–40)	41 (40–42)	41 (39–43)	43 (42–44)	43 (41–45)
*p* * ‘H’ vs. ‘A’	*p* ^†^ < 0.001; *p* ^‡^ < 0.001						*p* ^†^ < 0.001; *p* ^‡^ < 0.001					
*p* * ’H’ vs. ‘I’	*p* ^†^ = 0.061; *p* ^‡^ = 0.063						*p* ^†^ = 0.056; *p* ^‡^ = 0.059					
*p* * ’I’ vs. ‘A’	*p* ^†^ = 0.051; *p* ^‡^ = 0.049						*p* ^†^ = 0.054; *p* ^‡^ = 0.055					
WP-VAS M0-Median (IQR)	41 (38–44)	41 (39–43)	43 (41–45)	43 (41–45)	45 (42–48)	45 (41–49)	48 (46–50)	48 (47–49)	50 (47–53)	50 (46–54)	52 (50–54)	53 (50–56)
*p* * ‘H’ vs. ‘A’	*p* ^†^ < 0.001; *p* ^‡^ < 0.001						*p* ^†^ < 0.001; *p* ^‡^ < 0.001					
*p* * ’H’ vs. ‘I’	*p* ^†^ = 0.051; *p* ^‡^ = 0.053						*p* ^†^ = 0.046; *p* ^‡^ = 0.048					
*p* * ’I’ vs. ‘A’	*p* ^†^ = 0.047; *p* ^‡^ = 0.049						*p* ^†^ = 0.052; *p* ^‡^ = 0.054					
s-CTX-I M0- Median (IQR)	520 (460–600)	525 (465–600)	590 (510–670)	594 (514–680)	650 (600–700)	655 (610–700)	560 (500–620)	564 (510–620)	645 (600–700)	654 (604–700)	715 (650–780)	720 (660–790)
*p* * ‘H’ vs. ‘A’	*p* ^†^ < 0.001; *p* ^‡^ < 0.001						*p* ^†^ < 0.001; *p* ^‡^ < 0.001					
*p* * ’H’ vs. ‘I’	*p* ^†^ = 0.56; *p* ^‡^ = 0.059						*p* ^†^ = 0.051; *p* ^‡^ = 0.053					
*p* * ’I’ vs. ‘A’	*p* ^†^ = 0.049; *p* ^‡^ = 0.051						*p* ^†^ = 0.047; *p* ^‡^ = 0.049					
u-CTX-II M0-Median (IQR)	346 (300–390)	354 (304–394)	378 (330–415)	382 (334–420)	456 (416–496)	464 (420–500)	378 (318–438)	384 (324–442)	418 (350–478)	423 (353–483)	476 (426–526)	484 (430–530)
*p* * ‘H’ vs. ‘A’	*p* ^†^ < 0.001; *p* ^‡^ < 0.001						*p* ^†^ < 0.001; *p* ^‡^ < 0.001					
*p* * ’H’ vs. ‘I’	*p* ^†^ = 0.061; *p* ^‡^ = 0.063						*p* ^†^ = 0.056; *p* ^‡^ = 0.059					
*p* * ’I’ vs. ‘A’	*p* ^†^ = 0.049; *p* ^‡^ = 0.053						*p* ^†^ = 0.047; *p* ^‡^ = 0.049					
TH-BMD M0-Median (IQR)	0.8 (0.6–1.0)	0.8 (0.7–0.9)	0.6 (0.5–0.7)	0.6 (0.5–0.65)	0.45 (0.39–0.51)	0.45 (0.38–0.51)	0.7 (0.63–0.80)	0.7 (0.61–0.80)	0.5 (0.43–0.60)	0.5 (0.41–0.61)	0.35 (0.32–0.38)	0.35 (0.33–0.37)
*p* * ‘H’ vs. ‘A’	*p* ^†^ < 0.001; *p* ^‡^ < 0.001						*p* ^†^ < 0.001; *p* ^‡^ < 0.001					
*p* * ’H’ vs. ‘I’	*p* ^†^ = 0.065; *p* ^‡^ = 0.069						*p* ^†^ = 0.055; *p* ^‡^ = 0.059					
*p* * ’I’ vs. ‘A’	*p* ^†^ = 0.055; *p* ^‡^ = 0.058						*p* ^†^ = 0.049; *p* ^‡^ = 0.051					
mJSW M0-Median (IQR)	4.5 (4.4–4.6)	4.5 (4.3–4.7)	4.4 (4.0–4.8)	4.4 (4.1–4.7)	4.3 (4.0–4.6)	4.3 (4.1–4.5)	3.7 (3.6–3.8)	3.7 (3.5–3.9)	3.5 (3.4–3.6)	3.5 (3.3–3.7)	3.3 (3.1–3.5)	3.3 (3.0–3.6)
*p* * ‘H’ vs. ‘A’	*p* ^†^ = 0.060; *p* ^‡^ = 0.061						*p* ^†^ = 0.068; *p* ^‡^ = 0.069					
*p* * ’H’ vs. ‘I’	*p* ^†^ = 0.171; *p* ^‡^ = 0.173						*p* ^†^ = 0.163; *p* ^‡^ = 0.165					
*p* * ’I’ vs. ‘A’	*p* ^†^ = 0.149; *p* ^‡^ = 0.152						*p* ^†^ = 0.149; *p* ^‡^ = 0.153					

SC—standard of care (control) group; SG—study group; WOMAC-C—WOMAC function scale; WP-VAS—pain at walking by visual analogue scale; s-CTX-I—serum- beta-isomerized carboxy-terminal cross-linking telopeptide of type I collagen, the levels are presented as nanogram per milliliter; u-CTX-II—urine-C-terminal-crosslinking telopeptides of collagen type II, levels are presented as corrected concentrations of uCTX-II for urinary creatinine concentration, as ng/mmol Cr.; TH-BMD—total hip BMD; mJSW—median joint space width; *—Kruskal–Wallis test with Dunn–Bonferroni post hoc analysis; ^#^—each study group consisted of 25 subjects; *p* ^†^—*p*-values from the comparisons between the different radiographic models in the SC group; *p* ^‡^—*p*-values from the comparisons between the different radiographic models in the SG group.

**Table 4:** Changes in the clinical and radiographic parameters in the CG and SG groups over time.

Month Group	M0 (Baseline)	M12	M24	M36	M48	M60	M72	M78
**CG**								
**K/L-II”H”**								
**WP-VAS**	41 (38–44)	43 (41–45)	48 (46–49)	53 (51–54)	59 (57–60)	64 (62–65)	69 (68–70)	Conversion to THR-M78
**WOMAC-C**	32 (31–33)	34 (31–36)	38 (37–39)	43 (42–44)	49 (48–50)	55 (54–56)	60 (59–61)	
**s-CTH-I**	520 (460–600)	525 (460–610)	530 (470–610)	540 (480–620)	560 (500–640)	600 (540–680)	680 (620–740)	
**u-CTX-II**	346 (300–390)	348 (300–394)	350 (305–395)	355 (310–400)	365 (320–420)	385 (350–440)	425 (395–475)	
**TB-BMD**	1.5 (1.3–1.7)	1.4 (1.3–1.5)	1.3 (1.2–1.4)	1.2 (1.1–1.3)	1.1 (1.0–1.2)	1.0 (0.9–1.1)	0.9 (0.8–1.0)	
**mJSW**	4.4 (3.9–4.9)	3.9 (3.8–4.2)	3.3 (3.2–3.6)	2.6 (2.5–2.9)	1.8 (1.8–2.1)	1.1 (1.0–1.1)	0.5 (0.4–0.6)	
**K/L-II” I”**								
**WP-VAS**	43 (42–44)	46 (45–48)	51 (50–53)	57 (56–58)	62 (62–64)	69 (68–71)	Conversion to THR-M66	
**WOMAC-C**	33 (33–35)	36 (35–38)	41 (41–43)	47 (46–49)	53 (52–55)	59 (58–61)		
**s-CTH-I**	590 (510–670)	600 (520–680)	610 (530–690)	630 (550–710)	670 (590–750)	750 (670–830)		
**u-CTX-II**	378 (330–415)	382 (335–420)	390 (340–430)	400 (350–440)	420 (370–460)	440 (410–500)		
**TB-BMD**	1.4 (1.3–1.5)	1.3 (1.2–1.4)	1.2 (1.1–1.3)	1.1 (1.0–1.2)	1.0 (0.9–1.1)	0.9 (0.8–1.0)		
**mJSW**	4.3 (4.0–4.6)	3.8 (3.7–4.0)	3.1 (3.1–3.4)	2.4 (2.4–2.7)	1.7 (1.6–1.9)	0.8 (0.8–1.0)		
**K/L-II” A”**								
**WP-VAS**	45 (44–47)	50 (49–52)	55 (55–57)	61 (60–63)	68 (67–70)	Conversion to THR-M54		
**WOMAC-C**	36 (35–37)	40 (39–42)	45 (45–47)	51 (51–53)	58 (57–60)			
**s-CTH-I**	650 (600–700)	670 (620–720)	710 (660–760)	780 (720–840)	940 (880–990)			
**u-CTX-II**	456 (416–496)	460 (420–500)	475 (435–520)	505 (465–550)	570 (530–610)			
**TB-BMD**	1.3 (1.2–1.4)	1.2 (1.1–1.3)	1.1 (1.0–1.2)	1.0 (0.9–1.1)	0.9 (0.8–1.0)			
**mJSW**	4.2 (3.8–4.6)	3.6 (3.5–3.9)	2.9 (2.9–3.2)	2.2 (2.1–2.4)	1.3 (1.3–1.6)			
**K/L-III”H”**								
**WP-VAS**	48 (47–49)	48 (47–50)	54 (54–56)	61 (61–63)	69 (68–71)	Conversion to THR-M54		
**WOMAC-C**	38 (37–39)	38 (37–40)	45 (44–47)	52 (51–54)	59 (58–61)			
**s-CTH-I**	560 (500–620)	570 (510–630)	590 (530–650)	630 (570–690)	750 (690–810)			
**u-CTX-II**	378 (318–438)	384 (320–440)	395 (330–450)	420 (350–470)	480 (410–550)			
**TB-BMD**	1.4 (1.3–1.5)	1.3 (1.2–1.4)	1.2 (1.1–1.3)	1.1 (1.0–1.2)	1.0 (0.9–1.1)			
**mJSW**	3.6 (3.1–4.1)	3.0 (2.9–3.3)	2.3 (2.3–2.6)	1.6 (1.5–1.8)	0.8 (0.8–0.9)			
**K/L-III” I”**								
**WP-VAS**	50 (49–52)	51 (51–54)	58 (57–61)	66 (65–69)	Conversion to THR-M42			
**WOMAC-C**	41 (40–42)	42 (41–44)	49 (48–50)	56 (55–58)				
**s-CTH-I**	645 (600–700)	660 (620–715)	690 (650–730)	750 (710–800)				
**u-CTX-II**	418 (350–478)	425 (360–485)	440 (380–500)	480 (420–540)				
**TB-BMD**	1.3 (1.2–1.4)	1.2 (1.1–1.3)	1.1 (1.0–1.2)	1.0 (0.9–1.1)				
**mJSW**	3.5 (3.0–4.0)	2.9 (2.7–3.2)	2.1 (2.0–2.4)	1.2 (1.2–1.5)				
**K/L-III” A”**				THR-M36				
**WP-VAS**	52 (52–54)	54 (55–56)	60 (60–62)	72 (74–75)				
**WOMAC-C**	43 (42–44)	45 (44–46)	52 (51–53)	64 (63–65)				
**s-CTH-I**	715 (650–780)	745 (695–800)	820 (770–870)	950 (905–995)				
**u-CTX-II**	476 (426–526)	490 (440–540)	520 (470–570)	590 (540–640)				
**TB-BMD**	1.2 (1.1–1.3)	1.1 (1.0–1.2)	0.9 (0.8–1.0)	0.5 (05–0.6)				
**mJSW**	3.4 (2.9–3.9)	2.6 (2.6–2.9)	1.7 (1.6–1.8)	0.5 (0.5–0.6)				
**SG**								
**K/L-II”H”**								Conversion to THR-M78
**WP-VAS**	41 (40–42)	37 (34–38)	34 (32–36)	34 (32–36)	38 (37–39)	47 (46–49)	67 (66–69)	
**WOMAC-C**	31 (31–33)	27 (26–28)	24 (23–26)	25 (24–27)	28 (27–29)	38 (37–39)	58 (57–59)	
**s-CTH-I**	525 (465–600)	500 (440–675)	490 (420–560)	480 (410–550)	520 (450–590)	590 (520–650)	690 (620–750)	
**u-CTX-II**	354 (304–394)	350 (300–390)	354 (300–390)	360 (310–400)	370 (320–420)	390 (340–440)	430 (370–470)	
**TB-BMD**	1.5 (1.4–1.6)	1.5 (1.4–1.6)	1.6 (1.4–1.6)	1.6 (1.5–1.7)	1.7 (1.6–1.8)	1.6 (1.6–1.8)	1.5 (1.4–1.6)	
**mJSW**	4.4 (4.0–4.8)	3.9 (3.8–4.2)	3.4 (3.3–3.7)	2.9 (2.8–3.1)	2.3 (2.3–2.5)	1.6 (1.5–1.7)	0.7 (0.7–0.9)	
**K/L-II” I”**								Conversion to THR-M78
**WP-VAS**	43 (42–44)	38 (36–40) ^#^	33 (32–34) *	33 (31–34) *	38 (36–40) ^#^	48 (47–50)	67 (66–69)	
**WOMAC-C**	34 (33–35)	29 (28–30) ^#^	24 (23–25) *	24 (22–25) *	29 (28–30) ^#^	39 (38–40)	58 (57–59)	
**s-CTH-I**	594 (514–680)	545 (465–725)	450 (390–550)	350 (300–400)	440 (390–490)	550 (500–600)	700 (650–750)	
**u-CTX-II**	382 (334–420)	370 (320–400)	340 (290–430)	290 (240–380)	330 (280–420)	380 (340–420)	450 (410–490)	
**TB-BMD**	1.4 (1.3–1.5)	1.6 (1.3–1.5)	1.7 (1.4–1.6)	1.7 (1.5–1.7)	1.7 (1.6–1.8)	1.6 (1.6–1.8)	1.5 (1.4–1.6)	
**mJSW**	4.3 (3.9–4.7)	3.8 (3.8–4.1)	3.3 (3.3–3.5)	2.9 (2.8–3.0)	2.3 (2.2–2.4)	1.6 (1.5–1.7)	0.8 (0.7–0.9)	
**K/L-II” A”**							Conversion to THR-M66	
**WP-VAS**	45 (44–48)	39 (38–40) ^#^	35 (34–36) *	35 (34–37) *	40 (40–42) ^#^	62 (60–64)		
**WOMAC-C**	36 (35–37)	29 (28–30) ^#^	26 (24–27) *	25 (24–27) *	31 (30–32) ^#^	52 (51–54)		
**s-CTH-I**	655 (610–700)	500 (450–550)	410 (360–470)	330 (290–370)	420 (370–470)	650 (590–710)		
**u-CTX-II**	464 (420–500)	415 (370–460)	370 (320–420)	280 (240–320)	340 (290–430)	420 (360–480)		
**TB-BMD**	1.3 (1.2–1.4)	1.5 (1.2–1.4)	1.7 (1.3–1.5)	1.7 (1.4–1.6)	1.7 (1.5–1.7)	1.5 (1.4–1.6)		
**mJSW**	4.2 (3.8–4.6)	3.7 (3.7–4.0)	3.2 (3.2–3.4)	2.6 (2.6–2.9)	2.0 (2.0–2.3)	1.4 (1.4–1.6)		
**K/L-III”H”**						Conversion to THR-M54		
**WP-VAS**	48 (47–50)	45 (44–47)	49 (48–50)	56 (55–58)	67 (65–68)			
**WOMAC-C**	38 (37–40)	35 (34–37)	39 (38–40)	46 (45–48)	58 (57–59)			
**s-CTH-I**	564 (510–620)	560 (500–610)	550 (500–600)	530 (470–590)	680 (620–740)			
**u-CTX-II**	384 (324–442)	380 (320–440)	370 (310–430)	360 (300–420)	430 (390–470)			
**TB-BMD**	1.4 (1.3–1.5)	1.4 (1.3–1.5)	1.4 (1.3–1.5)	1.5 (1.4–1.6)	1.5 (1.4–1.6)			
**mJSW**	3.6 (3.2–4.0)	3.1 (3.0–3.4)	2.3 (2.0–2.3)	1.6 (1.4–1.6)	0.8 (0.7–0.9)			
**K/L-III” I”**						Conversion to THR-M54		
**WP-VAS**	50 (48–52)	46 (45–48) ^#^	50 (490–51)	57 (56–58)	67 (65–68)			
**WOMAC-C**	40 (39–42)	36 (35–38) ^#^	41 (40–42)	47 (56–59)	59 (59–62)			
**s-CTH-I**	654 (604–700)	600 (540–660)	520 (470–570)	460 (400–520)	650 (590–710)			
**u-CTX-II**	423 (353–483)	410 (370–450)	380 (320–420)	350 (290–410)	450 (390–510)			
**TB-BMD**	1.3 (1.2–1.4)	1.4 (1.2–1.4)	1.5 (1.3–1.5)	1.6 (1.4–1.6)	1.6 (1.4–1.6)			
**mJSW**	3.5 (3.1–3.9)	2.9 (2.8–3.1)	2.2 (2.1–2.3)	1.6 (1.5–1.6)	0.8 (0.7–0.9)			
**K/L-III” A”**					Conversion to THR-M42			
**WP-VAS**	53 (52–55)	50 (49–51) ^#^	56 (55–58)	68 (67–70)				
**WOMAC-C**	43 (42–45)	40 (39–41) ^#^	46 (45–48)	58 (47–60)				
**s-CTH-I**	720 (660–790)	590 (540–640)	500 (440–560)	550 (490–610)				
**u-CTX-II**	484 (430–530)	455 (400–510)	440 (380–500)	450 (390–510)				
**TB-BMD**	1.2 (1.1–1.3)	1.4 (1.1–1.3)	1.5 (1.2–1.4)	1.6 (1.4–1.6)				
**mJSW**	3.4 (3.0–3.8)	2.7 (2.6–2.8)	1.9 (1.7–1.9)	0.9 (0.9–1.0)				

Each group consisted of 25 subjects; CG—control group; SG—study group; WP—pain at walking; VAS—visual analogue scale -100 mm.; WOMAC-C—WOMAC-function scale; s-CTX-I—serum-beta-isomerized carboxy-terminal cross-linking telopeptide of type I collagen in nanogram per milliliter; u-CTX-II—urine-C-terminal crosslinking telopeptides of collagen type II, presented as corrected concentrations of uCTX-II for urinary creatinine concentration in ng/mmol Cr. mJSW—mean joint space width. Values associated with the OARSI responses are marked with * and these with MCII responses, with ^#^.

**Table 5:** Within-group comparisons in clinical parameters (WP/F) between the RMs of the same RG at different time points.

Treatment Groups ^#^	Time (Months) for the Occurrence of SSD in Clinical Parameters (WP/F)
**SC**	
K/L-II’H’ vs. K/L-II’A’	M0 (*p* < 0.001) *
K/L-II’H’ vs. K/L-II’I’	M12(*p* = 0.044) *
K/L-II’I’ vs. K/L-II’A’	M12 (*p* = 0.033) *
K/L-III’H’ vs. K/L-III’A’	M0 (*p* < 0.001) *
K/L-III’H’ vs. K/L-III’I’	M12 (*p* = 0.044) *
K/L-III’I’ vs. K/L-III’A’	M12 (*p* = 0.033) *
**SG**	
K/L-II’H’ vs. K/L-II’A’	M0 (*p* < 0.001) *
K/L-II’H’ vs. K/L-II’I’	No SSD were detected between M0-M78 (*p* > 0.05) *
K/L-II’I’ vs. K/L-II’A’	M60 (*p* < 0.001) *
K/L-III’H’ vs. K/L-III’A’	M0 (*p* < 0.001) *
K/L-III’H’ vs. K/L-III’I’	No SSD were detected between M0-M54 (THR) (*p* > 0.05) *
K/L-III’I’ vs. K/L-III’A’	M36 (*p* = 0.044) *

K/L—radiographic grade according to Kellgren–Lawrence; ‘A’/’I’/’H’—radiographic models (atrophic/intermediate/hypertrophic); CG—control group; SG—study group; WP—pain at walking; F—function (WOMAC-C); ^#^—Each subgroup consists of 25 subjects; *—Kruskal–Wallis test was applied.

**Table 6:** Within-group comparisons of the radiological indicators (JSN/mJSW) between RMs of the same RG and on the same treatment.

X-ray Grade	Applied Treatment ^#^	X-ray Model	JSN-M12 Median (IQR) mm./yearly	*p* * Value	mJSW-M36 Median (IQR) mm.	*p* * Value
K/L-II	SC	‘H’	0.55 (0.50–0.60)	‘H’ vs. ‘A’; *p* < 0.001	2.6 (2.5–2.9)	‘H’ vs. ‘A’; *p* < 0.001
		‘I’	0.57 (0.54–0.60)	‘H’ vs. ‘I’; *p* = 0.044	2.4 (2.4–2.7)	‘H’ vs. ‘I’; *p* = 0.044
		‘A’	0.61 (0.60–0.62)	‘I’ vs. ‘A’; *p* = 0.022	2.2 (2.1–2.3)	‘I’ vs. ‘A’; *p* = 0.044
	SG	‘H’	0.55 (0.50–0.60)	‘H’ vs. ‘A’; *p* = 0.059	2.6 (2.5–3.0)	‘H’ vs. ‘A’; *p* = 1.0
		‘I’	0.56 (0.52–0.60)	‘H’ vs. ‘I’; *p* = 0.796	2.6 (2.4–2.8)	‘H’ vs. ‘I’; *p* = 1.0
		‘A’	0.50 (0.45–0.55)	‘I’ vs. ‘A’; *p* = 0.049	2.6 (2.3–2.9)	‘I’ vs. ‘A’; *p* = 1.0
K/L-III	SC	‘H’	0.62 (0.59–0.65)	‘H’ vs. ‘A’; *p* < 0.001	1.6 (1.5–1.8)	‘H’ vs. ‘A’; *p* < 0.001
		‘I’	0.67 (0.67–0.70)	‘H’ vs. ‘I’; *p* = 0.021	1.2 (1.2–1.5)	‘H’ vs. ‘I’; *p* = 0.023
		‘A’	0.80 (0.70–0.90)	‘I’ vs. ‘A’; *p* < 0.001	0.5 (0.4–0.6)	‘I’ vs. ‘A’; *p* < 0.001
	SG	‘H’	0.61 (0.58–0.64)	‘H’ vs. ‘A’; *p* = 0.767	1.7 (1.5–1.9)	‘H’ vs. ‘A’; *p* < 0.001
		‘I’	0.62 (0.59–0.65)	‘H’ vs. ‘I’; *p* = 0.796	1.3 (1.2–1.6)	‘H’ vs. ‘I’; *p* = 0.049
		‘A’	0.62 (0.59–0.65)	‘I’ vs. ‘A’; *p* = 1.0	0.8 (0.7–0.9)	‘I’ vs. ‘A’; *p* = 0.045

K/L—radiographic grade according to Kellgren–Lawrence; ‘A’/’I’/’H’—radiographic models (atrophic/intermediate/hypertrophic); CG—control group; SG—study group; JSN—joint space narrowing; mJSW—mean joint space width; ^#^—Each subgroup consists of 25 subjects; *—Kruskal–Wallis test was applied.

**Table 7:** Between-group comparisons of the changes in the radiological indicators JSN at M12 and mJSW at M36.

X-ray Grade	X-ray Model	Applied Treatment ^#^	mJSN-M12 Median (IQR)	*p*-Value *	mJSW-M36 Median (IQR)	*p*-Value *
**K/L II**	**H**	SC	0.55 (0.50–0.60)	SG vs. SC; *p* = 1.0	2.6 (2.5–2.9)	SG vs. SC; *p* = 1.0
		SG	0.55 (0.50–0.60)		2.6 (2.5–3.0)	
	**I**	SC	0.57 (0.54–0.60)	SG vs. SC; *p* = 1.0	2.4 (2.4–2.7)	SG vs. SC; *p* = 0.23
		SG	0.56 (0.52–0.60)		2.5 (2.4–2.8)	
	**A**	SC	0.61 (0.60–0.62)	SG vs. SC; *p* < 0.001	2.2 (2.1–2.3)	SG vs. SC; *p* < 0.001
		SG	0.50 (0.45–0.55)		2.6 (2.4–2.8)	
**K/L III**	**H**	SC	0.62 (0.59–0.65)	SG vs. SC; *p* = 1.0	1.6 (1.5–1.8)	SG vs. SC; *p* = 1.0
		SG	0.61 (0.58–0.64)		1.7 (1.5–1.9)	
	**I**	SC	0.67 (0.67–0.70)	SG vs. SC; *p* =1.0	1.2 (1.2–1.5)	SG vs. SC; *p* = 0.21
		SG	0.62 (0.59–0.65)		1.3 (1.2–1.6)	
	**A**	SC	0.80 (0.70–0.90)	SG vs. SC; *p* < 0.001	0.5 (0.4–0.6)	SG vs. SC; *p* = 0.03
		SG	0.62 (0.59–0.65)		0.8 (0.7–0.9)	

The time points M12 and M36 were selected as the time of first occurrence (M12) and presence at follow-up in all subgroups (M36). CG—control group; SG—study group; ‘A’/’I’/’H’—atrophic/intermediate/hypertrophic radiological models of HOA; JSN—joint space narrowing (millimeter/yearly); mJSW—median joint space width (millimeter); *—Kruskal–Wallis test was applied; ^#^—Each group consisted of 25 subjects.

**Table 8:** Between-group comparisons (SC vs. SG) regarding the length of time before THR had to be performed.

X-ray Pattern	Treatment ^#^	Treatment 1 vs. Treatment 2 tTHR -Median (Months)	*p*-Value *
K/L III-‘A’	SG vs. CG	42 vs. 36	<0.001
K/L III-‘I’	SG vs. CG	54 vs. 42	<0.001
K/L III-‘H’	SG vs. CG	54 vs. 54	1.000
K/L-II-‘A’	SG vs. CG	66 vs. 54	<0.001
K/L-II’I’	SG vs. CG	78 vs. 66	<0.001
K/L-II’H’	SG vs. CG	78 vs. 78	1.000

CG—control group; SG—study group; THR—total hip replacement; ^#^—Each study group consisted of 25 subjects; *—Kruskal–Wallis test was applied.

**Table 9:** Multiple logistic regression to determine the factors associated with radiographic progression in HOA.

Factors	OR	95% CI of OR		*p*-Value
		Lower	Upper	
**FM-BMD ^b^**	6.561	2.590	16.617	<0.001
**TH-BMD ^b^**	6.495	2.688	15.696	<0.001
**HAL ^a^**	2.212	1.182	4.141	0.013
**NSA ^b^**	2.377	1.171	4.826	0.017
**CSA ^b^**	4.038	1.863	8.752	<0.001
**CSMI ^b^**	2.724	1.301	5.706	0.008
**MNW ^b^**	1.099	0.614	1.967	0.751
**FN-CT ^b^**	1.578	0.812	3.104	0.177
**FS-CT ^b^**	1.236	0.603	2.533	0.563
**SM ^b^**	3.431	1.617	7.280	0.001
**BR ^a^**	1.833	1.012	3.321	0.045

FM-BMD—femoral neck-BMD; TH-BMD—total hip-BMD; HAL—hip axis length; NSA—neck shaft angle; CSA—cross-sectional area; CSMI—cross-sectional moment of inertia; MNW—minimal neck width; FN-CT—cortical thickness of femoral neck; FS-CT—cortical thickness of femoral shaft; SM –section modulus; BR—buckling ratio; ^a^—increased values of these variables were associated with accelerated progression; ^b^—decreased values of these variables were associated with accelerated progression.

## Data Availability

Raw data were generated at University Hospital “Pulmed”. Derived data supporting the findings of the study are available from the corresponding author (LS), on request.
